# Epidemic Spread on Weighted Networks

**DOI:** 10.1371/journal.pcbi.1003352

**Published:** 2013-12-12

**Authors:** Christel Kamp, Mathieu Moslonka-Lefebvre, Samuel Alizon

**Affiliations:** 1 Paul-Ehrlich-Institut, Federal Institute for Vaccines and Biomedicines, Langen, Germany; 2 INRA, UR 0341 Mathématiques et Informatique Appliquées, Jouy-en-Josas, France; 3 AgroParisTech, F-75005 Paris, France; 4 Laboratoire MIVEGEC (UMR CNRS 5290, IRD 224, UM1, UM2), Montpellier, France; Imperial College London, United Kingdom

## Abstract

The contact structure between hosts shapes disease spread. Most network-based models used in epidemiology tend to ignore heterogeneity in the weighting of contacts between two individuals. However, this assumption is known to be at odds with the data for many networks (e.g. sexual contact networks) and to have a critical influence on epidemics' behavior. One of the reasons why models usually ignore heterogeneity in transmission is that we currently lack tools to analyze weighted networks, such that most studies rely on numerical simulations. Here, we present a novel framework to estimate key epidemiological variables, such as the rate of early epidemic expansion (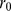) and the basic reproductive ratio (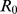), from joint probability distributions of number of partners (contacts) and number of interaction events through which contacts are weighted. These distributions are much easier to infer than the exact shape of the network, which makes the approach widely applicable. The framework also allows for a derivation of the full time course of epidemic prevalence and contact behaviour, which we validate with numerical simulations on networks. Overall, incorporating more realistic contact networks into epidemiological models can improve our understanding of the emergence and spread of infectious diseases.

## Author Summary

Understanding how infectious diseases spread has public health and ecological implications. The contact structure between hosts strongly affects this spread. However, most studies assume that all types of contacts are identical, when in reality some individuals interact more strongly than others. This is particularly striking for sexual-contact networks, where the number of sex acts is not identical for all partnerships. This heterogeneity in activity can either speed up or slow down epidemic spread depending on how strongly the individuals' number of contacts coincides with their activity. There are two limitations to current frameworks that can explain the lack of studies on weighted networks. First, analytical results are difficult to obtain, which requires numerical simulations. Second, inferring weighted networks from survey data is extremely difficult. Here, we present a novel framework that allows to alleviate these two limitations. Building on configuration type network epidemic approaches, we manage to capture disease spread on weighted networks from the distribution of the number of contacts and distribution of the number of interaction events (e.g. sex acts). This allows us to derive analytical estimates for the epidemic threshold and the rate of spread of the disease. It also allows us to readily incorporate survey data, as illustrated in this study with data from the National Survey of Sexual Attitudes and Lifestyles (NATSAL) carried out in the UK.

## Introduction

Contact structure between hosts is known to have a key influence on disease spread [1]. A striking result is for instance that the more heterogeneous the contact network is, i.e. the higher the variance in the number of contacts per individual, the more rapid the initial disease spread.

One way to capture contact structure is to use a network [2]. Such contact networks are typically described by a square binary adjacency matrix, where each term on the *i*th line and *j*th column can take the value 0 or 1 to indicate respectively the absence or the presence of a contact between individuals *i* and *j*. Contact networks are widely used because they possess several convenient properties, one of which being that the dominant eigenvalue of the adjacency matrix is an indicator of the initial propagation speed of an infectious disease spreading on this network [3], [4].

The main limitation of contact networks is that their exact shape is often difficult to infer. This is why there is a continuous effort to predict disease spread from network summary statistics that are easier to estimate, such as the distribution of the number of contacts (degrees). For instance, the number of secondary infections generated by a typical infected host in a fully susceptible population, i.e. the basic reproductive number 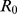 [1], scales with the ratio of the second moment 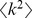 and first moment (mean) 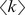 of the distribution in the number of contacts *k*. This result holds both for static networks (denoted 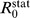) [5] as well as for fully mixed, dynamic networks (denoted 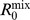) [6], [7] with
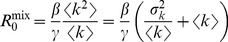
(1a)

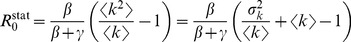
(1b)where 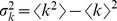 is the variance of the distribution of the number of contacts. The static case corresponds to networks in which the identity of contacts is fixed (as approximatively seen in sexual contact networks) and the fully mixed dynamic case corresponds to a situation in which individuals update their contacts dynamically in a fully mixed fashion within the population (as approximatively seen in airborne infections).


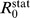
 and 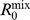 represent the lower and upper bounds of the basic reproductive ratio [8] for SIR epidemics on random networks if individuals transmit the infection at a rate *β* and recover from the infection at a rate *γ*. On both static and dynamic heterogeneous networks with a large or even diverging variance in the distribution of the number of contacts, epidemics die out only for very small or even vanishing transmission rates *β*.

One of the typical key assumptions epidemiological models on networks make to obtain such elegant expressions for 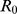 is that the transmission rate is the same between all pairs of individuals. This is materialized by the fact that all the edges of the contact matrix have a weight of 0 or 1. This is known to be a simplifying assumption [9]. A well-studied example related to infectious diseases is that of sexual contact networks, where the number of sex acts per unit of time is not constant in all partnerships [10]–[12]. More generally, the number of interaction events (which correspond to potential transmission events) may vary among contact pairs and is likely to decrease with the number of contacts an individual has (see also Figure S3). Simplifying the reality is commendable but the problem is that tampering with the weighting of the network has been shown to lead to the loss of important epidemiological properties of heterogeneous unweighted networks, such as the low value of the epidemiological threshold or the negative correlation between the epidemiological threshold value and network size [13]. To summarize, although contact networks appear to be ‘scale free’ in structure, they might not exhibit the properties one might expect from this structure.

An increasing number of studies in epidemiology point to the importance of considering weighted networks. Some examples include the spread of sexually-transmitted infections [13], disease transmission in sheep flocks [14], respiratory diseases of humans [15] or general infectious diseases of human spreading on a social contact network [16] or on airline connection networks [17]. Several more conceptual studies have also been published in the theoretical physics literature (e.g. [2], [18]–[20]). Most of these studies have in common that they use weighted networks and resort to (heavy) numerical simulations. Indeed, contrary to unweighted networks, we lack analytical frameworks to analyze epidemic spread on weighted networks.

**Figure 1:**
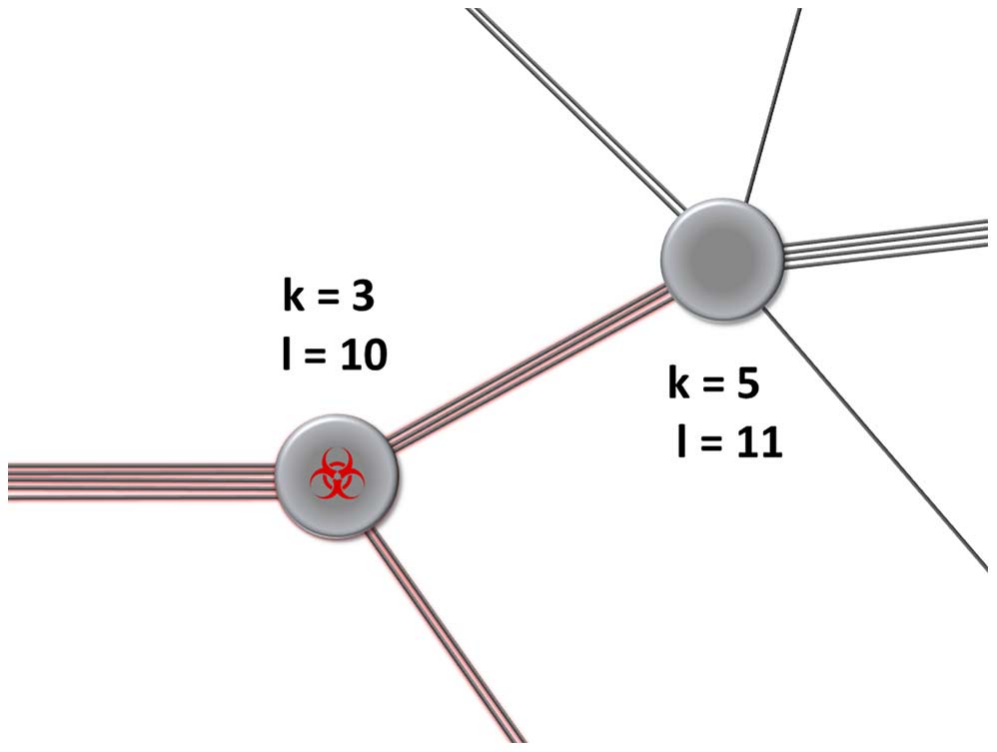
Weighting between contacts. Both the number of contacts that an individual maintains and the weight that (s)he assigns to each contact are relevant for the spread of an infectious agent. Here, each individual has *l* interaction events that (s)he can distribute among his/her *k* contacts. On the scale of the transmission network, these are modelled by the joint probability distribution 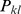 to find an individual with *k* contacts and *l* interaction events per time interval.

Here, we present an original framework, which builds on configuration type network epidemic approaches [21], [22] that offers an alternative to simulating epidemics on full networks. It allows to model the dynamics of a disease spreading on a weighted network and to estimate key epidemiological variables from the network's properties. The framework provides us with explicit expressions for the rate of early epidemic expansion (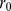) and the basic reproductive ratio (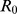) of the infection without requiring strong simplifying assumptions regarding epidemiological processes or the distribution of weights on the contact network. It also allows for a derivation of the full time course of epidemic prevalence and contact behaviour of susceptible, infected and recovered individuals (in terms of the probability generating functions – PGFs – of the degree distributions). As sketched in Fig. 1, the parametrisation is done in a general fashion using the joint probability distribution 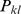 of an individual to have *k* contacts among which (s)he randomly distributes *l* interaction events. We validate our analytical results using numerical simulations on networks.

Importantly, since this framework relies on summary statistics of the network and does not require knowledge of the exact shape of the network, it can be parametrized using large scale survey data. The network information we lose by using these summary statistics requires that we make the assumption that there is no assortativity between individuals in our framework. However, we show that even with these assumption we can approximate epidemic dynamics better than with non weighted networks.

## Materials and Methods

### The configuration model

Individuals have *k* contacts and *l* interaction events per time interval, which are distributed among their *k* contacts (*l* is sometimes also referred to as the strength of the node [9]).

The model is broadly applicable as it can be parameterised through any joint probability distribution of the number of partners (*k*) and number of interaction events (*l*). Such a joint probability distribution 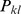 can be written as the product 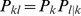, 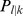 being the probability distribution of the number of interaction events per time *l* given that the individual has *k* contacts. If 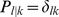, where 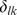 is 1 if *l* = *k* and 0 otherwise, we are then back to a ‘classical’ network case, with an exact linear dependency between the number of contacts and the number of interaction events (for a detailed discussion, see Text S1, Section D, *The recovery of the classical equations in the linear case*). Our framework can capture more general situations by explicitly choosing 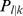.

Our analytical approximation assumes that an individual distributes his/her *l* interaction events multinomially among his/her *k* contacts, which are static and are randomly assigned as in configuration models [21], [22]. This individual is infected at a rate proportional to his/her average number of interaction events with *i* infected contacts among his/her total *k* contacts (so 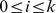). This averaging implies the choice of a time scale for the number of interaction events such that 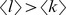 (the network shape is assumed to be constant over the time period considered so the number of contacts is not affected by the time scale). The analytical approach only relies on the nodes' statistics and does not consider the constraints for half-contacts to match half-contacts with similar weight, whereas in reality, the weight on a link between two nodes should be the same for the two nodes. This could lead to an unrealistic network segregation for some artificial networks where 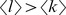. This network segregation can affect epidemic dynamics in these networks in a way that is not seen in the analytical approach *per se* but can be considered through corrections in the analytical approach as we discuss later on.

### The epidemiological model

A susceptible individual becomes infected at a rate proportional to the number of interaction events per time interval (*l*), the transmission probability per interaction event of the pathogen (*β*) and the probability for each of his/her contacts to points to an infected individual (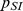). The population dynamics of susceptible, infected and recovered individuals with *k* contacts and *l* interaction events per time interval (denoted 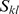, 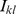 and 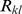 respectively) are thus captured by the following set of differential equations:
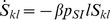
(2a)

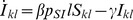
(2b)

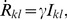
(3c)where *γ* is the rate at which hosts recover and become immune to subsequent infection (see Table 1). This setting can capture lifelong infections if *γ* = 0.

**Table 1:** Notations used in the study.

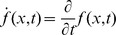	partial derivative of function *f* with respect to *t*
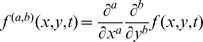	partial derivative of function *f a* times with respect to *x* and *b* times with respect to *y*
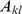	number of individuals in group *A* with *k* contacts and *l* (potential) transmission events (per time interval)
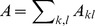	number of individuals in group *A*
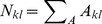	number of individuals with *k* contacts and *l* transmission events (per time interval)
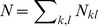	total number of individuals
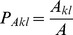	probability for an individual in group *A* to have *k* contacts and *l* transmission events per time interval
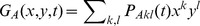	probability generating function (PGF) of 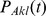
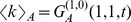	average number of contacts of *A* individuals
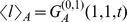	average number of transmission events per time interval of *A* individuals
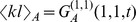	average number of contacts times transmission events per time interval of *A* individuals
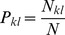	probability for an individual to have *k* contacts and *l* (potential) transmission events per time interval
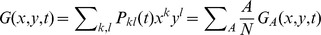	probability generating function (PGF) of 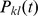
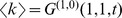	average number of contacts of individuals
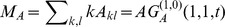	number of links coming from *A* individuals
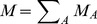	number of links
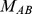	number of links coming from *A* individuals and pointing to *B* individuals
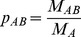	probability for a link starting from an *A* individual to point to an *B* individual

*A*,*B* correspond to epidemic stages, i.e. *S*, *I*, *R* for susceptible, infected, recovered.

The dynamics of the total number of susceptible, infected and recovered individuals can be obtained by summing over *k* and *l* in equation system 2. This leads to:
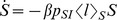
(3a)

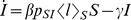
(3b)

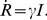
(3c)where 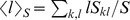 is the average number of interaction events per time a susceptible individual has.

To close the equation system (2)–(3), we need expressions for the temporal dynamics of the 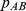, i.e. the probabilities for a status *A* individual's contact to be with an individual in state *B*. These can be derived through a careful assessment of the links/contacts among susceptible and infected individuals over the course of an epidemic. This means following the dynamics of the joint probability distribution to find *k* contacts and *l* interaction events per time among susceptible individuals 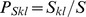 through its PGF, denoted 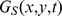. The temporal dynamics of 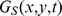 can be calculated by observing that the dynamics of the corresponding joint probability distribution of contacts and interaction events of susceptible individuals are governed by the equation 
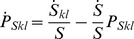
. Hence, we close equation system (2)–(3) with the following equations:
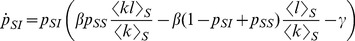
(4a)

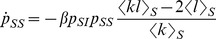
(4b)

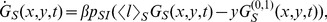
(4c)where 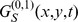 is the partial derivative of 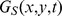 with respect to *y*. Note that 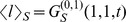 and 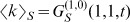. For furthers details about the terms in this equation system, see Table 1.

The set of differential equations describing the epidemic process is derived by careful bookkeeping of the links along which an infectious agent spreads (a detailed derivation is provided in Text S1, Section A, *Equations for the epidemic model on weighted networks*).

### Analytical results and their validation

#### Distributions used

To validate the analytical approach, we generated four types of networks corresponding to the combinations of homogeneous and heterogeneous behaviour in the number of contacts *k* and the number of interaction events *l* as well as the corresponding networks with a linear dependency between *k* and *l*. We studied the spread of an infectious agent on these artificial networks using the analytical approach by plugging the corresponding joint probability generating functions into equations (3) and (4).

More precisely, we consider combinations of Poisson and power law distributions for the number of contacts *k* and interaction events per time *l*. As we neglect isolated hosts, we use the Poisson distribution 
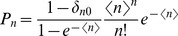
 with support for 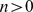 and probability generating function 
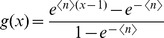
 and power law distributions with exponent *λ* and cut-off *κ*, 
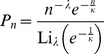
 and 
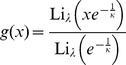
 (normalisation through the Polylogarithm 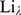) for homogeneous and heterogeneous behaviour, respectively. If the joint probability distribution 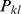 is given by the product 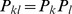 of independent distributions with PGFs 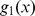 and 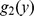, their joint PGF 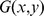 is also given by the product 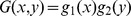. In the linear case with 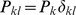, the PGF 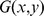 is given by 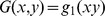.

#### Generating networks from PGF

Networks were generated by assigning each host a number *k* of ‘half-contacts’ (stubs) and *l* interaction events per time interval drawn from the distribution 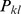. Each host then shared his/her interaction events equi-probably at random (i.e. multinomially) among his/her *k* contacts. 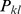 was chosen to satisfy 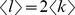 corresponding to a timescale in which a host has on average 2 interaction events per contact.

The matching of half-contacts (stubs) was done at random but by respecting their assigned number of interaction events per contact. The problem is that not all probability distribution 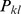 can be realised topologically within a network. Indeed, weighting of interactions between contacts can impose strong constraints on network topology [23]. The fact that contacts can only occur between stubs with the same weight can lead to network segregation and assortative effects that are not seen in the analytical approach *per se* due to its node-centric view (see Text S1, Section E, *Network segregation and the limiting case* 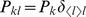). It is even possible to devise joint probability distributions 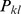 that cannot be represented through a network topology (although this is not a problem for 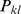 empirically derived from realised networks). In simulated networks, this necessity for exact matching segregates the network into subnetworks, which can show assortativity either with respect to the degree of connected nodes or with respect to their edge weights.

#### Correcting for assortativity

If the number of interaction events per time and per contact is large (i.e. if 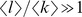), hosts nearly equi-distribute their interaction events among all their contacts due to convergence under the law of large numbers. Decreasing 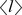 yields a more realistic network, as this introduces some variability in the number of interaction events among a host's contacts, which offers more flexibility in the assignment of interaction events on short time scales and reduces assortative effects.

*Weight assortativity* arises in networks that have a (nearly) constant number of contacts *k* per individual and where the number of interaction events *l* is distributed in a heterogeneous way, thus leading to an early expansion among the most highly active individuals. This can accelerate the initial expansion of an epidemic but, at the same time, it also constrains disease spread compared to what one could expect from the analytical approach. Alternatively, the network can also segregate with respect to the number of contacts an individual holds (i.e. *contact or degree assortativity*). An extreme case can be observed when a (nearly) constant number of interaction events has to be distributed among a heterogeneous number of contacts. This leads to a (near) isolation of individuals with single contacts from the epidemic process (see Text S1, Section E, *Network segregation and the limiting case* 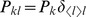).

We can introduce some tolerance in ‘negotiating’ the number of interaction events per contact. Another way to deal with weight and degree correlations between neighbouring individuals is to drop the assumption that weights are multinomially distributed among an individual's contact. Indeed, heterogeneous weight distributions among an individual's contacts can reduce correlations among neighbouring individuals' degrees and weights [23]. However, this cannot be done without changing 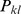 to an empirical distribution 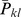, while at the same time deviations might arise from the analytical approach as the assumption of multinomial distribution of weights is violated.

### Simulations on weighted networks

Networks for simulation are obtained by first generating 10,000 nodes with *k* half-contacts (stubs) and *l* interaction events as drawn from the probability distribution 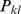. The *l* interaction events a node has are then distributed multinomially among its *k* stubs. Stubs are randomly matched together, with matches being rejected if the weights of the stubs differ by more than one interaction event. In addition matches are rejected if they differ by more than 10% of the smaller weight involved to avoid biases in nodes with few links. If stubs with non-identical weights are matched, the contact is assigned the mean weight randomly rounded to the next integer. This results in the empirical distribution 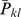 as mentioned in the previous subsection.

We use the Gillespie direct algorithm [24] to run stochastic SIR epidemics on continuous time. For each susceptible node *i*, transmission occurs at rate 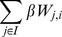, where *β* is the probability of transmission per sex act, *W* is the weighted adjacency matrix listing the number of interaction events per time unit between all pairs of nodes, and *I* is the set of infected nodes connected to node *i*. Infected nodes recover at rate *γ*. For each case analyzed, 20 nodes were initially infected uniformly at random in a population of 10,000 and 100 replicate simulations were carried out over each of 20 replicate networks.

### Derivation of 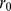 and 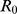


The derivation of the early exponential growth rate 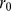 is based on the observation that the rate of epidemic expansion as described by equations (3a–3c) is proportional to the number of links/contacts between susceptible and infected individuals 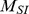, i.e. 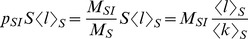, with 
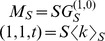
 being the number of contacts of susceptible individuals.

As there is no explicit expression for 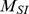 we have to rely on approximate equations that are valid in the early stages of an epidemic. In the mean field approximation, 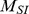 is approximated by the number of infected individuals (*I*) and the average number of contacts per individual found originally in the total population (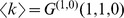). As soon as the epidemic is set, i.e. once we are beyond the mean field approximation relying on a randomly picked node, 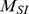 is given by the product of *I* and a slightly more sophisticated estimate of the number of contacts of infected hosts than in the mean field approximation. More precisely, each infected node contributes to 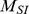 by the average excess degree of a recently infected node chosen proportional to its number of interaction events 
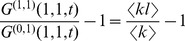
 (for details, see Text S1, Section A, *Equations for the epidemic model on weighted networks*). This means that the contact the infection has spread from is discounted and that all ‘new’ contacts are assumed to still be susceptible in the early phase of an epidemic. In order to correctly estimate 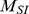 in the full chain of early infections, it is necessary to discount not only the contact from which an individual got infected but also the contact along which the epidemic spreads further. This results in 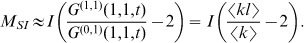.

Altogether we have,
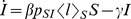


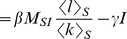



We also have 
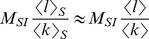
 where the approximation holds in the early phase of the epidemic.

If we assume that 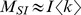, then
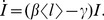
(5)If we assume that 
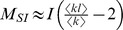
, then
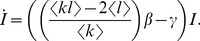
(6)The exponential growth rate of the infected population in equation (6) corresponds to 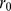. Approximation (5) corresponds to a ‘mean field approximation’ representing the neighbourhood of a randomly picked node, i.e. not a node picked according to its number of interaction events per time interval. Approximation (6) considers that an infected individual has been picked with a probability proportionally to its number of interaction events per time interval. The doubling time 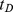 can be derived from the early exponential growth rate 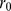 as 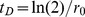.

The basic reproductive ratio 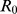 is the average number of secondary infections that a typical infected host produces in a fully susceptible population. As for SIR models on classical random networks, it is derived by first evaluating the distribution of excess contacts of a typically infected host, i.e. the probability for a node chosen according to its number of interaction events per time (*l*) to have *k* excess contacts. This probability is 
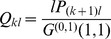
. 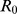 is calculated in Text S1 (Section C, *The basic reproductive ratio* 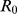) as the number of infections that spread along these excess contacts before recovery of the typically infected host.

## Results

### Validation of the analytical model with simulations on networks

In order to test our analytical model, we consider epidemiological dynamics taking place on artificial networks on which we release the constraints found in ‘classical’/unweighted networks by assuming that the number of interaction events *l* an individual has does not necessarily increase linearly with his/her number of partners *k*. To create these networks, we used combinations of Poisson and power law distributions for the number of contacts *k* and interaction events per time *l*. This allowed us to introduce arbitrary combinations of homogeneous or heterogeneous behaviour in the way contacts are made and in the number of interaction events established, that may be either independent or dependent (as in the linear case).

**Figure 2:**
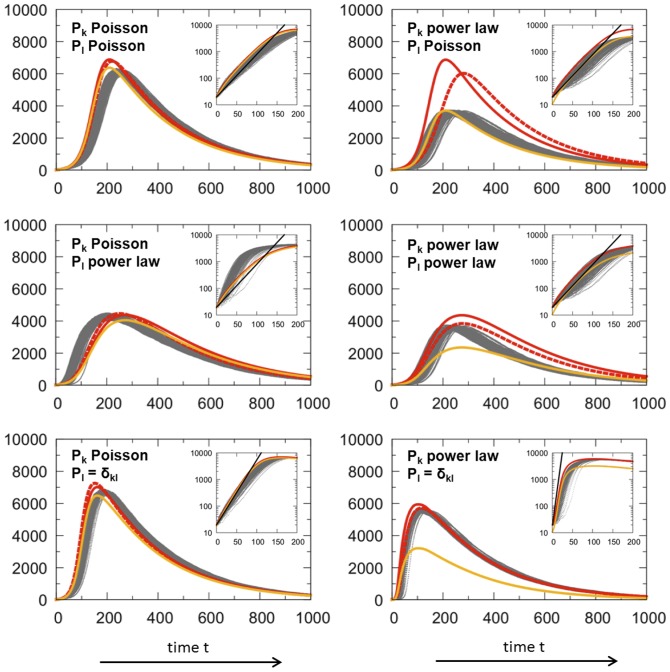
Dynamics of the number of infected hosts (*I*) during epidemic spreading on different types of networks. The distributions in the number of contacts (*k*) and interaction events per time (*l*) are either homogeneous (Poisson) or heterogeneous (power law). For the number of interaction events, we also show the linear case in which *l* is strictly proportional to the number of contacts *k*, i.e. 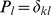. *k* and *l* are drawn from joint distributions 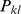 with 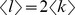 (except for the analytical 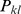 model's linear case where 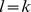 being compensated by a double transmission rate). The figures show the epidemic prevalence *I* as the outcome of the simulation runs (grey, dotted lines), of the the numerical solution of the analytical model with 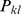 (red, solid line) and 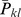 (red, dashed line). In addition, we show the epidemic prevalence when excluding individuals with only one contact (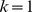) which is relevant for epidemics on networks with heterogeneous numbers of contacts including many individuals with 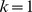 in combination with a (nearly) constant number of interaction events, as realised through a Poisson distribution (orange line, cf. specifically power law, Poisson). Parameters chosen correspond to 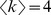 (Poisson case: 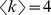, 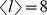, power law case: 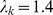, 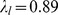, 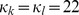). Epidemiological parameters are *β* = 0.01 (0.02 for the analytical 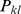 model's linear case), *γ* = 0.004 in arbitrary units and *I*(0) = 20. The insets show the same data for the early epidemic expansion in logarithmic scale showing early exponential growth according to 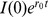 (black line) with 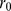 from Table 2.

To validate the model, we compared the epidemic prevalence (*I*) from repeated simulation runs with the results derived from the analytical approach using the probability generating functions corresponding to 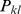 and 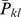, 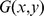 and 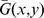. (Note that 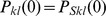, 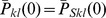, 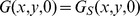 and 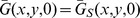.) The epidemiological dynamics are summarised in Fig. 2. In addition of the analytical approach for 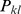 (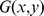), 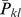 (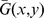), we also show an approximation (applied to 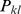), in which we exclude individuals with one contact. The latter is relevant for networks with heterogeneous number of contacts and (nearly) constant number of interaction events per individual (contact or degree assortativity). Quantitative measures to assess the level of discrepancy between simulations and approximations are provided in Text S1 (Section F, *Agreement between approximations and simulations*) and Table S2. The derivation of analytical expressions for the error (e.g. a 95% confidence interval) is likely to be an extremely difficult task as these expressions should consider the complex implications on epidemic dynamics that are caused by deviations in the network topology from those of random networks. The simplest way might be to use numerical simulations to estimate the magnitude of the error for given networks.

Overall, the analytical approach matches the simulation results well if the constraints imposed by 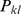 on the specific networks topology are properly taken into account. The assortativity correction (in orange) is most relevant for a heterogeneous distribution of contacts (*k* follows a power law) and a homogeneous distribution of interaction events (*l* is Poisson distributed). This is because the strongest constraints on network topology are expected to occur with these distributions (Fig. 2, top panel, right side). On the contrary, for more homogeneous networks (Fig. 2, left panel), the assortativity correction is less needed. In the linear case, the correction is not relevant (Fig. 2, bottom panel).

A particularly interesting observation is that epidemics spread slower on networks with heterogeneous contacts if the network is weighted than if the number of interaction events scales linearly with the number of contacts for each individual (i.e. ‘classical’ linear networks). This is particularly true in networks where the average weight per contact is inversely proportional to the number of contacts an individual has. In our simulations, this effect is the clearest when 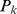 follows a power law distribution and 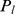 a Poisson distribution (Fig. 2 top panel, right side).

### Capturing epidemic characteristics (expressions for *r*_0_ and *R*_0_)

There are different ways to assess the initial propagation of an infectious agent in an otherwise fully susceptible population. One possibility is to estimate the initial exponential growth rate in the number of infected individuals (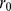). Another possibility consists in estimating the number of secondary cases created by a newly infected host in a fully susceptible population, which is classically referred to as the basic reproductive ratio 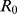 [1].

Subtle effects arise depending on whether we choose the neighbourhood of a random individual as a reference or the neighbourhood of a ‘typically’ infected individual. The first case corresponds to what is usually referred to as a ‘mean field approximation’ and captures well the very first infection events. The second case (using a ‘typical’ infected individual) is more appropriate to capture the next stages of early epidemic expansion because it accounts for the fact that spatial structure has been sensed or set by the epidemic process. We thus use it for the derivation of 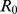.

The expressions for 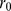 and 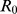 for the SIR model are shown in Table 2 and are derived in details in the Materials and Methods and in Text S1, respectively. Note that these do not involve any approximation beyond those implied in the model's assumptions, i.e. they are exact within the model framework. The derivation for 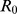 when 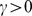 is the only case that requires some further approximations to obtain an explicit formula (see Text S1, Section C, *The basic reproductive ratio* 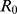).

**Table 2:** 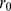
 vs. 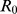.

	Early epidemic growth rate 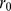	Basic reproductive ratio 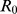
Epidemic expansion from randomly picked index case, (mean field approximation) 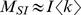	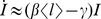 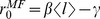	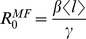
Early epidemic expansion, structure set by epidemic, 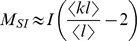	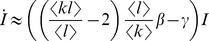 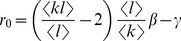	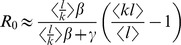

*β* is the transmission probability, *γ* the recovery rate, 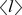 the average number of interaction events an individual has, 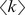 the average number of contacts per individual, 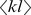 is the second moment of the joint probability distribution 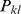 and 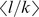 is the average number of interaction events per contact. Note that the equation for 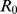 is exact for 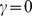.

In the expression for 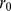 and 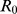, all the occurrences of the transmission probability (*β*) are weighted by the number of interaction events per contact. This makes sense because this corresponds to a transmission rate. Note that there is a slight difference between 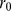 and 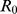 because in the former we have the ratio of the means (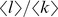), whereas in the latter we have mean of the ratios (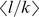). This is due to the fact that the averaging is done at a different step in the calculations.

More interestingly, the expressions for 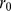 and 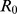 both scale with the second moment 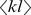 of the joint probability distribution 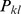. This implies that the number of contacts (*k*) and the interaction events (*l*) an individual maintains equally affect epidemic spread. At the same time the correlation between these quantities is relevant to model rapid epidemic spread: for epidemic control, targeting individuals with most contacts or interaction events can prove to be much less efficient than targeting those who maximise both. The formulae in Table 2 are generalisations of formula 1b, which corresponds to the linear case where the number of interaction events scales with the number of contacts a person maintains, i.e. 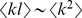. Note that earlier approaches on weighted networks correspond to the fully mixed situation in formula 1 in which *k* is interpreted as the number of interaction events [6].

Figure 2 shows epidemic expansion in different types of simulated networks (grey, dotted lines) in comparison with analytical approximations (red and orange lines). The insets in logarithmic scale show that the exponential growth rate 
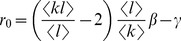
 calculated for the early epidemic expansion approximates well the simulation and analytical results. The early exponential growth rate 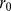 can slightly underestimate the dynamics if the contact network is homogeneous (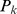 is Poisson distributed), while the distribution of interaction events is heterogeneous (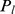 follows a power law). This underestimation is due to the fact that weight assortativity generates a subnetwork of individuals with many interaction events, which speeds up epidemic expansion in its early phase (Fig. 2, middle panel, left side).

### Application of the model to epidemiological data

The knowledge of transmission networks along which an infectious agent can spread within a host population is of great importance to public health. These networks might be hard to assess for air-borne infections because they are very dynamic [25], [26] but easier to infer for sexually transmitted infections (STI) because they are more static. Such sexual contact networks have been surveyed in many studies covering homosexual as well as heterosexual populations and different societal contexts [27]–[29] to understand and prevent the spread of STI. The National Survey of Sexual Attitudes and Lifestyles (NATSAL [28]) provides detailed data on the situation in the United Kingdom, including distributions in the number of sexual partners (*k*) and sex acts (interaction events, *l*) a person has within certain time frames.

**Figure 3:**
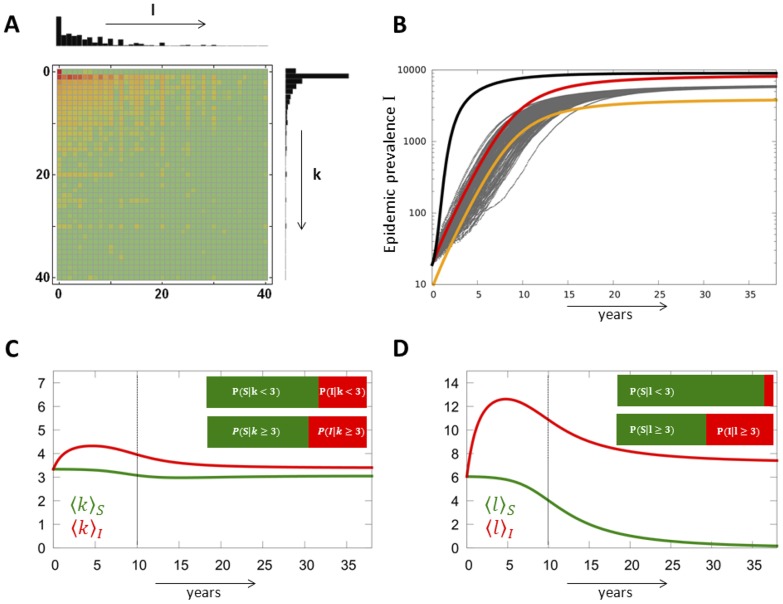
Disease spread on a network inferred from data. A) Characteristics of the heterosexual contact network inferred from the NATSAL contact tracing study [28]. The network shows a heterogeneous joint probability distribution 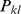, which is the probability for an individual to have *k* contacts during the last 5 years and *l* sex acts during the last 4 weeks (higher values of 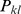 are in red and lower values are in green). This heterogeneity is also seen for the marginal distributions 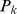 (on the right) and 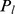 (on the top). B) Dynamics of an SI epidemic spreading on an unweighted (black line) or a weighted sexual contact network. The results of simulations on the weighted network are in grey, the approximations of our model are in red or in orange for the case with assortativity correction. The network has been reduced to nodes with *k*>0 and transmission probability per sex act is *β* = 0.01. C) Dynamics of the average number of contacts 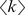 of susceptible (in green) and infected individuals (in red) over the course of an epidemic spreading on the weighted network. The inset shows the probability of a host to be susceptible or infected at *t* = 10 years conditioned to the number of contacts during the last 5 years (more or less than 3 contacts). D) Same as panel C but for the number of sex acts 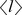. In Panels B, C and D, the weighting is done using the 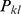 shown in panel A. Individuals with more contacts tend to be disproportionally infected (panel C). Individuals with more sex acts tend to be even more infected (panel D).

As shown in Fig. 3A, both the number of partners (contacts, *k*) and sex acts (interaction events, *l*) an individual has are heterogeneously distributed. However, their joint distribution 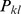 does not show a linear behaviour, implying that the number of sex acts *l* does not scale linearly with the number of partners *k* an individual has. This is also supported by Pearson's correlation coefficient which is 0.15, i.e. positive but not indicating a strong linear relationship between *k* and *l* (see also Supporting Figure S3). When they combine a linear relationship between number of partners and number of sex acts with the observed broad distributions of sexual contacts and sex acts, several models predict extremely rapid early epidemic expansion and an epidemic threshold that is potentially vanishing in the limit of infinite network size [5]–[8], [30], [31], as can be seen from equation (1).

In our ‘Validation’ section we have shown that, regarding the number of interaction events, a deviation from the linear behaviour decreases epidemic expansion and peak prevalence, especially for transmission networks that are characterized by a heterogeneous distribution in the number of contacts per individual *k* and regardless of whether the distribution of interaction events/sex acts is homogeneous (Poisson) or heterogeneous (power law). This is also reflected more quantitatively in the expression for 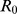, which is dominated by the second moment 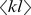 of 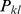. In other words, (as explained above when interpreting our approximations) the more the number of partners (*k*) correlates with their number of sex acts (*l*) the faster the early epidemic expansion.

These differences between weighted and unweighted networks are visible in Fig. 3, which shows the epidemic expansion of a susceptible-infected (SI) epidemic with transmission probability *β* = 0.01 per sex act in two scenarios. We chose to model SI dynamics by setting *γ* = 0 in order to simulate HIV spreading on a short time scale, but the model could be evaluated with analogous results for SIR dynamics (see Text S1, Section G, *Captions of the Supplementary Figures*, Figure S1). In the first scenario (black curve, Fig. 3B), the number of sex acts grows linearly with the number of contacts respecting the average number of sex acts (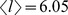 during 4 weeks). In the second scenario we use a weighted network, where the joint probability distribution 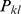 is obtained from the NATSAL data (red curve, Fig. 3B) and can be complemented by a correction for assortative effects (orange curve, Fig. 3B).

The exponential growth rates of the epidemics are 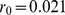 per year for the linear, unweighted, network and 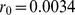 per year for the weighted network. This confirms that the ‘linear’ scenario supports faster epidemic expansion. The correction for assortative effects underestimates the epidemic prevalence in the network because in the NATSAL network heterogeneity in the number of contacts and interaction events does not lead to a strong network segregation, i.e. individuals with a single or few contacts are not isolated (see Fig. 2 and the case in which both 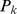 and 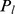 follow power law distributions). Although the survey data shown in Fig. 3A only provide us with a rough picture of the real transmission network and although relying on the number of partners during 5 years overestimates the number of concurrent partners, the data are sufficient to confirm a remarkable reduction in the speed of epidemic expansion when shifting from a classical unweighted transmission network towards a more realistic weighted transmission network. This finding is in particular consistent with an earlier simulation study on epidemic spreading along a network of homosexual contacts [13].

The framework can also be used to follow the dynamics in the epidemic subgroups and to identify risk groups along the course of the epidemics (see Text S1, Section B, *Conditional probabilities and risk groups*). Fig. 3C and D show that the average number of contacts 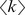 and sex acts 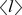 per individual increases early on in the infected population and decreases in the healthy population. This reflects the over-representation of some individuals among those being infected as sketched by the insets: during the epidemic (here shown at *t* = 10 years) the probability of being infected grows with the number of contacts *k* an individual has, and even more so with the number of sex acts *l* (s)he has.

## Discussion

Network theory has broadened our understanding of the spread of infectious agents — or other entities such as information, money, travellers or goods — in complex settings. In their simplest form, network models do not consider that contacts may show variability in their transmission capacity. However, the probability of disease transmission along a contact strongly depends on the intensity of the contact, transportation links vary in their throughput and information may not be shared equally among all possible channels. Although earlier studies have shown that this weighting in terms of interactions between contacts has non-negligible impact on the spreading dynamics, the modelling of epidemics on weighted networks largely focuses on simulation studies [13], [16], [32], regular networks [33], mean field approximations [34], [35] or discrete time dynamics [20], [36]. Therefore, explicit expressions for epidemic characteristics such as the basic reproductive ratio 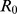 are available only in special cases.

It is possible to simplify the epidemiology by using a Reed-Frost model. For this, one needs to assume that infections take place in discrete time steps, with non-overlapping generations and that each infected individual recovers with certainty one time step after infection. These simplifications allow to assess outbreak probabilities using branching processes [20]. In this formalism, as shown in [36], 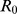, denoted 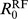, can be derived as the dominant eigenvalue of the mean offspring matrix (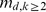), where 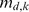 represents the expected number of individuals with *k* contacts that an individual with *d* contacts infects considering potentially degree-dependent network weights. Importantly, it is only because Britton *et al.* make strong simplifying assumptions in their model, such as the independence between network weights and nodes' degrees, that they can derive an explicit form of 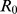. In contrast to our findings on NATSAL data, the Reed-Frost approach systematically predicts negative exponential growth rates of the epidemics for both scenarios (the network average of linear case is 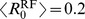 and that of survey data case is 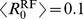). The discrepancy between our model and that of [20] stems from the implicit assumption of the Reed-Frost model with discrete time steps they use, which is that recovery occurs immediately after infection and therefore that 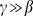.

We extend earlier results by developing a framework based on partial differential equations that allows to model continuous time SIR epidemic dynamics for general weighted networks defined through the joint probability distribution for an individual to have *k* contacts and *l* interaction events. From this we are able to derive the full epidemic dynamics in terms of the number of susceptible, infected and recovered individuals over time as well as explicit expressions for the basic reproductive ratio 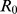 and the exponent of early epidemic expansion 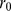. The application of the method to epidemics on artificial and empirically-motivated networks matches well with simulation results on these same networks. Moreover, it also stresses the impact of assortative effects introduced by contact weighting on epidemic dynamics; an aspect that will need closer attention in future research.

One limitation to our approach is due to potential errors in the inference of the network. There are known biases in the self-reporting of number of partners (with different trends between men and women [37]) and self-reported number of sex acts are likely to exhibit similar biases. One extension of this study would be to see how such noise in the network inference could affect epidemic spread. Our intuition is that the consequences should be less important than for non weighted networks because heterogeneity in the weights is already likely to dampen striking network properties in terms of disease spread [13].

As many earlier methods, ours analyses model disease spread on networks from a node centric summary statistics, by considering the number of contacts and transmission events per time. Therefore, it inherently neglects correlation between nodes. In other words there is no consideration of assortativity between individuals based on their number of contacts or transmission events per time. At the same time, individuals share their activity randomly among all their contacts (weights are homogeneously, or multinomially, distributed among edges that leave a node), which can enforce correlations among nodes in certain networks. Also clustering is observed in many contact networks [38] and this issue should be addressed in an extended version of our model.

Most analytical and numerical models predict disease spread on network using only one summary statistics, the distribution of the number of partners. We show that additional insights can be gained, while maintaining some analytical results, by including another summary statistics, such as the distribution of the number of sex acts knowing the number of partners. These data are easier to collect than full information of the contact network (especially for a weighted network), which makes our framework widely applicable. We demonstrate this applicability here using data from the NATSAL study conducted in the UK. We note that for some artificial distributions, our results begin to diverge from simulations on real networks. However, the framework has proven to be applicable for empirical distributions and analysis of more empirical data will allows us to further test the robustness of the method using more realistic assumptions.

## Supporting Information

Figure S1 Epidemic SIR dynamics on the network as presented in Fig. 3 of the main manuscript. Transmission probability per sex act is also *β* = 0.01 but recovery can occur at a rate *γ* = 0.004 per 4 weeks, i.e. parameters corresponding to Fig. 2 of the main manuscript. Different from the SI dynamics shown in Fig. 3 of the main manuscript hosts may recover and do not spread infection indefinitely.
(PNG)

Figure S2Epidemic incidence or rate of infection 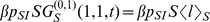 (cf. equation 3b) for SI dynamics (grey line) and SIR dynamics (dark grey line) on the network as presented in Fig. 3 of the main manuscript.(PNG)

Figure S3 Relationship between a person's total number of sex acts and number of partners derived from the NATSAL data. In Panel A, we plot the self-reported number of sex acts over the last 4 weeks *vs.* the self-reported number of sexual partners over the last 4 years. In Panel B, we plot the self-reported number of sex acts over the last 4 weeks *vs.* the self-reported number of sexual partners over the last 3 months. In Panel C, we plot the self-reported number of sex acts over the last 7 days *vs.* the self-reported number of sexual partners over the last 3 months. In all three cases, the data do not support a linear relationship (the number of sex acts per partner decreases with the number of partners/contacts).
(PNG)

Table S1 Model notations.
(PDF)

Table S2 Agreement between approximations and simulations.
(PDF)

Text S1 Supporting Information.
(PDF)
