# Supplementary figures and images for: Epidemic Spread on Weighted Networks
Source: PLoS Comput Biol. 2013 Dec 12;9(12):e1003352. doi: 10.1371/journal.pcbi.1003352 (PMC3861041; doi:10.1371/journal.pcbi.1003352)

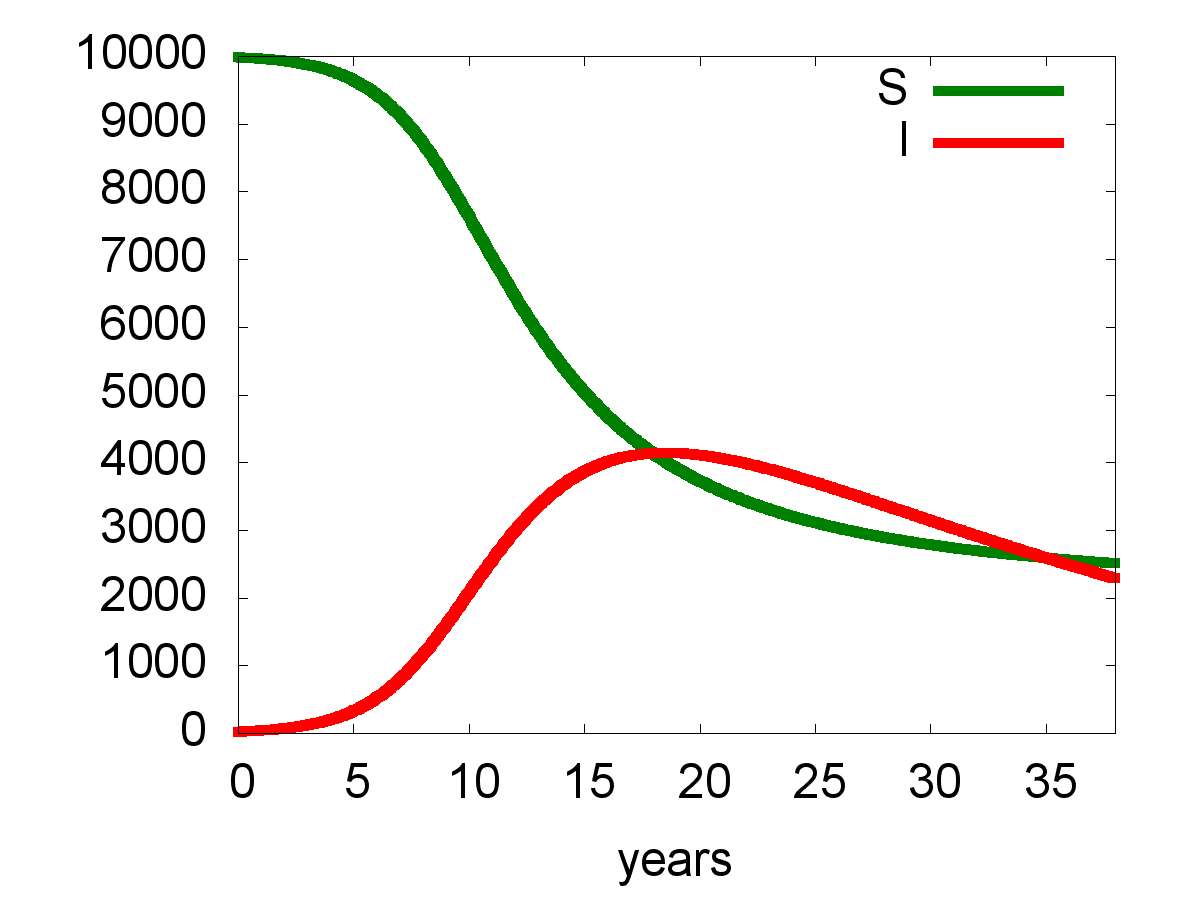

Supplement: Figure S1 — Epidemic SIR dynamics on the network as presented in Fig. 3 of the main manuscript. Transmission probability per sex act is also β = 0.01 but recovery can occur at a rate γ = 0.004 per 4 weeks, i.e. parameters corresponding to Fig. 2 of the main manuscript. Different from the SI dynamics shown in Fig. 3 of the main manuscript hosts may recover and do not spread infection indefinitely. (PNG) [file pcbi.1003352.s001.png]

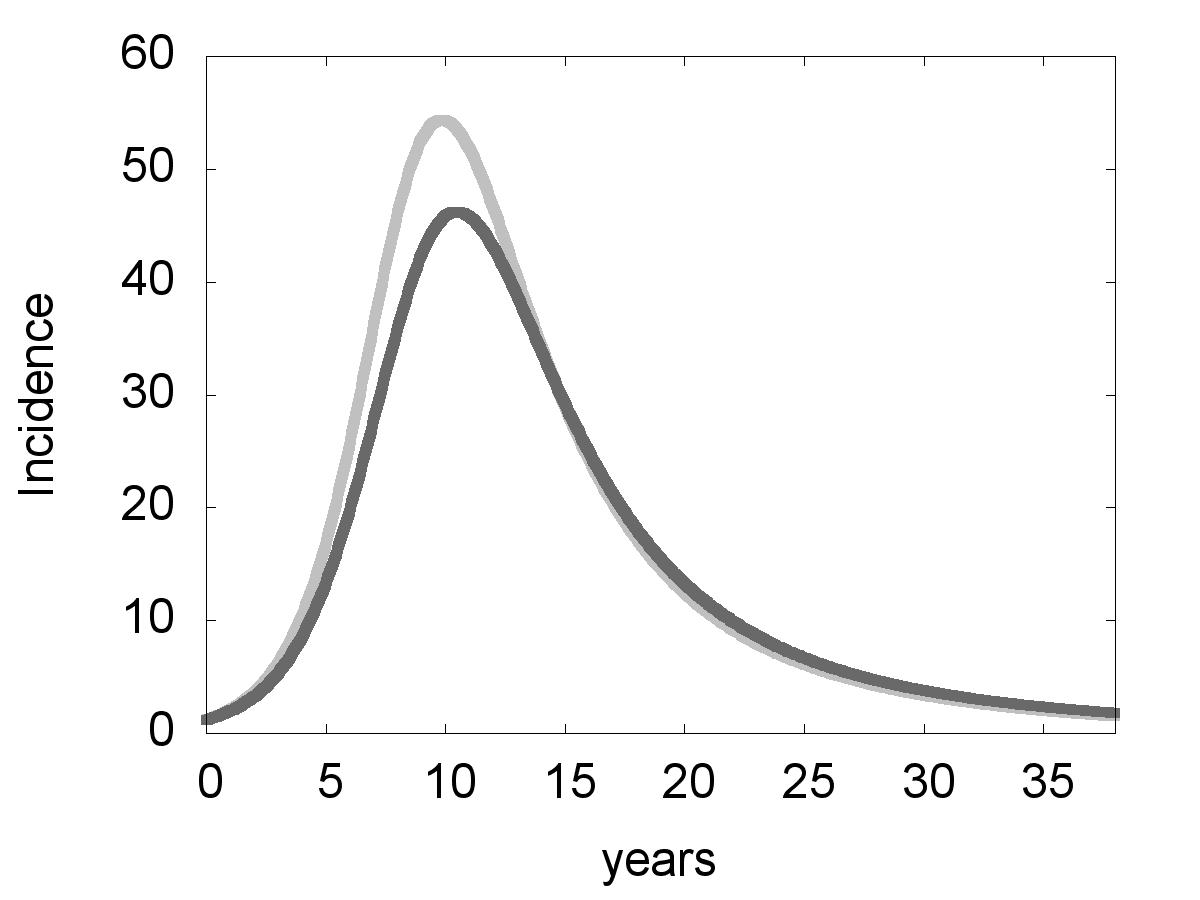

Supplement: Figure S2 — Epidemic incidence or rate of infection (cf. equation 3b) for SI dynamics (grey line) and SIR dynamics (dark grey line) on the network as presented in Fig. 3 of the main manuscript. (PNG) [file pcbi.1003352.s002.png]

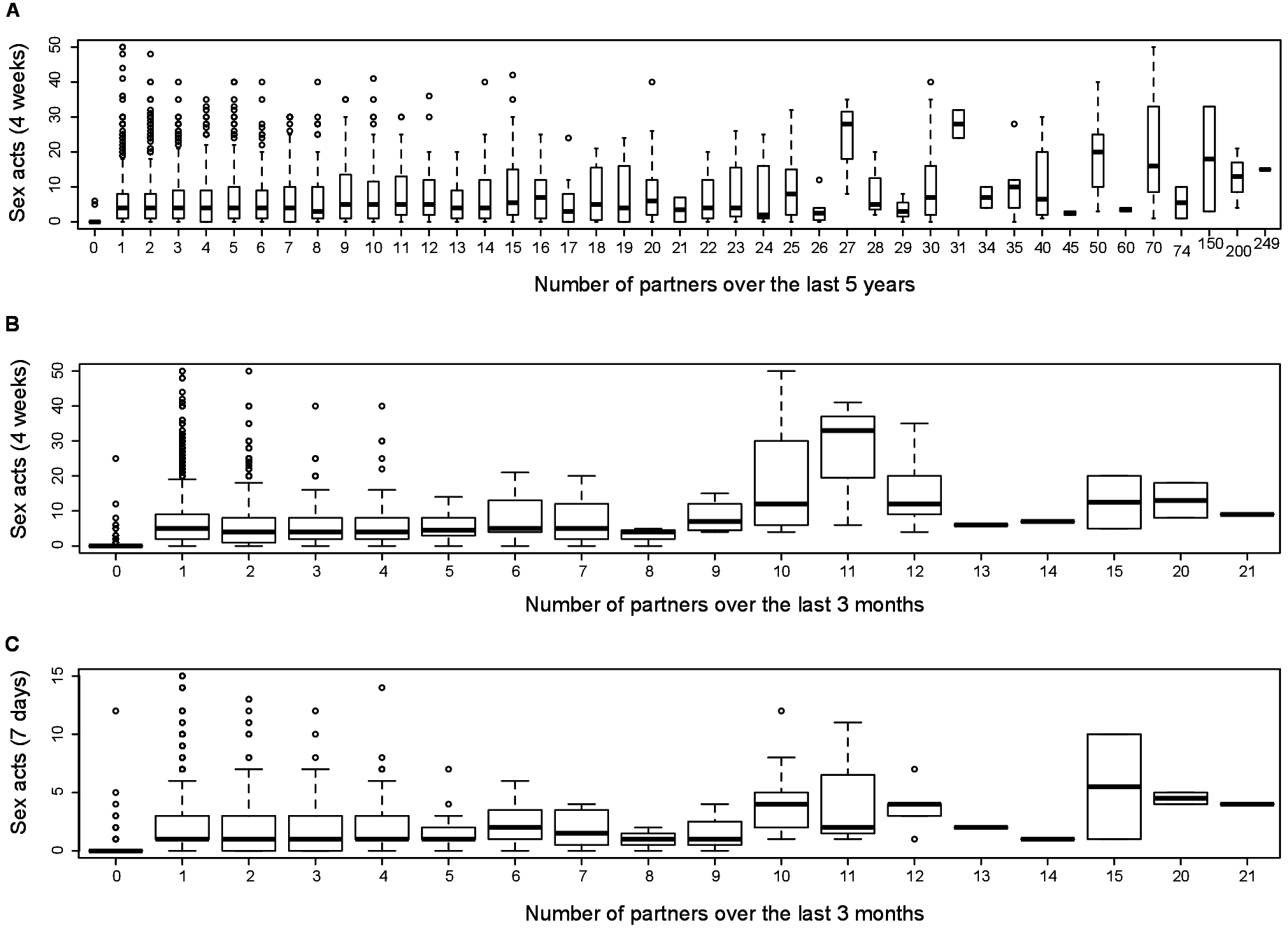

Supplement: Figure S3 — Relationship between a person's total number of sex acts and number of partners derived from the NATSAL data. In Panel A, we plot the self-reported number of sex acts over the last 4 weeks vs. the self-reported number of sexual partners over the last 4 years. In Panel B, we plot the self-reported number of sex acts over the last 4 weeks vs. the self-reported number of sexual partners over the last 3 months. In Panel C, we plot the self-reported number of sex acts over the last 7 days vs. the self-reported number of sexual partners over the last 3 months. In all three cases, the data do not support a linear relationship (the number of sex acts per partner decreases with the number of partners/contacts). (PNG) [file pcbi.1003352.s003.png]
