# Supplementary material for: Epidemic Spread on Weighted Networks
Source: PLoS Comput Biol. 2013 Dec 12;9(12):e1003352. doi: 10.1371/journal.pcbi.1003352 (PMC3861041; doi:10.1371/journal.pcbi.1003352)
Supplement: Table S1 — Model notations. (PDF) [file pcbi.1003352.s004.pdf]

Table S1: Model notations

$\dot{f}(x,t) = \frac{\partial}{\partial t} f(x,t)$	partial derivative of function f with respect to t
$f^{(a,b)}(x,y,t) = \frac{\partial^a}{\partial x^a}\frac{\partial^b}{\partial y^b} f(x,y,t)$	partial derivative of function f a times with respect to x and b times with respect to y
A_{kl}	number of individuals in group A with k contacts and l interaction events (per time interval)
$A = \sum_{k,l} A_{kl}$	number of individuals in group A
$N_{kl} = \sum_A A_{kl}$	number of individuals with k contacts and l interaction events (per time interval)
$N = \sum_{k,l} N_{kl}$	total number of individuals
$P_{Akl} = \frac{A_{kl}}{A}$	probability for an individual in group A to have k contacts and l interaction events per time interval
$G_A(x,y,t) = \sum_{k,l} P_{Akl}(t) x^k y^l$	probability generating function (PGF) of $P_{Akl}(t)$
$\langle k \rangle_A = G_A^{(1,0)}(1,1,t)$	average number of contacts of A individuals
$\langle l \rangle_A = G_A^{(0,1)}(1,1,t)$	average number of interaction events per time interval of A individuals
$\langle kl \rangle_A = G_A^{(1,1)}(1,1,t)$	average number of contacts times interaction events per time interval of A individuals
$P_{kl} = \frac{N_{kl}}{N}$	probability for an individual to have k contacts and l interaction events per time interval
$G(x,y,t) = \sum_{k,l} P_{kl}(t) x^k y^l$ $= \sum_A \frac{A}{N} G_A(x,y,t)$	probability generating function (PGF) of $P_{kl}(t)$
$\langle k \rangle = G^{(1,0)}(1,1,t)$	average number of contacts of individuals
$M_A = \sum_{k,l} k A_{kl} = A G_A^{(1,0)}(1,1,t)$	number of links coming from A individuals
$M = \sum_A M_A$	number of links
M_{AB}	number of links coming from A individuals and pointing to B individuals
$p_{AB} = \frac{M_{AB}}{M_A}$	probability for an undirected link starting from an A individual to point to an B individual

A,B correspond to epidemic stages, i.e. S, I, R for susceptible, infected, recovered
