# Supplementary material for: Epidemic Spread on Weighted Networks
Source: PLoS Comput Biol. 2013 Dec 12;9(12):e1003352. doi: 10.1371/journal.pcbi.1003352 (PMC3861041; doi:10.1371/journal.pcbi.1003352)
Supplement: Table S2 — Agreement between approximations and simulations. (PDF) [file pcbi.1003352.s005.pdf]

Table S2: Agreement between approximations and simulations

	type	P_k Poisson P_l Poisson	P_k Poisson P_l power	P_k Poisson $P_l = \delta_{kl}$	P_k power P_l Poisson	P_k power P_l power	P_k power $P_l = \delta_{kl}$
τ^*	y_{raw}	+21	-39	+13	+7	0	+9
	y_{rem}	+21	-39	+13	+7	0	+9
	y_{emp}	+12	-26	+19	-29	0	0
$\mathcal{C}^*$	y_{raw}	0.99	0.97	0.99	0.99	1.00	1.00
	y_{rem}	0.99	0.97	0.99	0.99	1.00	1.00
	y_{emp}	0.99	0.98	0.99	0.98	1.00	1.00
a	y_{raw}	1.06	1.02	1.01	1.78	1.47	1.05
	y_{rem}	0.99	0.95	0.94	0.96	0.80	0.57
	y_{emp}	1.07	1.01	1.02	1.65	1.26	1.02
p value	y_{raw}	< 0.001	< 0.001	< 0.001	< 0.001	< 0.001	< 0.001
	y_{rem}	< 0.001	< 0.001	< 0.001	< 0.001	< 0.001	< 0.001
	y_{emp}	< 0.001	< 0.001	< 0.001	< 0.001	< 0.001	< 0.001
R^2	y_{raw}	0.97	0.94	0.98	0.99	0.99	0.99
	y_{rem}	0.97	0.94	0.98	0.99	0.99	0.99
	y_{emp}	0.98	0.97	0.97	0.96	0.99	1.00

τ^* is the time lag that maximizes the cross-correlation coefficient between simulations and approximations ($\mathcal{C}$; the maximum of which is denoted $\mathcal{C}^*$). a, p-value and R^2 represent respectively the slope, p-value and percentage of variance explained associated with a linear regression of approximations against simulations. Based on our summaries, the match between simulations and approximations is maximal when $\tau^* = 0$, $\mathcal{C}^* = 1$, $a = 1$, p-value< 0.001 and $R^2 = 1$
