# Supplementary material for: Epidemic Spread on Weighted Networks
Source: PLoS Comput Biol. 2013 Dec 12;9(12):e1003352. doi: 10.1371/journal.pcbi.1003352 (PMC3861041; doi:10.1371/journal.pcbi.1003352)
Supplement: Text S1 — Supporting Information. (PDF) [file pcbi.1003352.s006.pdf]

Supporting Information for the manuscript "Epidemic spread on weighted networks"

Christel Kamp[1,*], Mathieu Moslonka-Lefebvre[2] and Samuel Alizon[3]

[1] Paul-Ehrlich-Institut, Federal Institute for Vaccines and Biomedicines, Paul-Ehrlich-Straße 51-59, 63225 Langen, Germany, Tel: +49 6103 77 3718, email: christel.kamp@pei.de

[2] INRA, UR 341 Mathématiques et Informatique Appliquées, 78350 Jouy-en-Josas, France ,Tel : +33 1 34 65 22 47, email: mathieu.moslonka-lefebvre@jouy.inra.fr

[3] Laboratoire MIVEGEC (UMR CNRS 5290, IRD 224, UM1, UM2), 911 avenue Agropolis, 34394 Montpellier Cedex 5, France, Tel : +33 4 67 41 64 36, email: samuel.alizon@cnrs.fr

* Corresponding author

Contents

A Equations for the epidemic model on weighted networks **3**

B Conditional probabilities and risk groups **8**

C The basic reproductive ratio R_0 **9**

D The recovery of the classical equations in the linear case $P_{kl} = P_k\delta_{kl}$ **11**

E Network segregation and the limiting case $P_{kl} = P_k\delta_{\langle l\rangle l}$ (constant case) **13**

F Agreement between approximations and simulations **14**

G Captions of the Supplementary Figures **16**

H Captions of the Supplementary Tables **17**

A Equations for the epidemic model on weighted networks

We consider an epidemic of a disease that is transmitted with probability β per interaction event and from which infected individuals recover at a rate γ. Susceptible individuals with k contacts and l interaction events per time interval are infected at a rate proportional to β, l and the probability that a susceptible individual's contact is made with an infected individual p_{SI}. This leads to the following equations for the evolution of the number of susceptible and infected individuals with k (infectious) contacts and l interaction events per time

$$\dot{S}_{kl} = -\beta p_{SI} l S_{kl} \quad (1a)$$

$$\dot{I}_{kl} = +\beta p_{SI} l S_{kl} - \gamma I_{kl} \quad (1b)$$

$$\dot{R}_{kl} = \gamma I_{kl}. \quad (1c)$$

A detailed overview of the model's notation and parameters is given in Table S1.

Adding up the contributions for all k and l introduces the average number of interaction events per time and susceptible individual $\langle l \rangle_S = \sum_l l P_{Skl} = \sum_l l \frac{S_{kl}}{S}$ into the equations. This average number can also be expressed in terms of probability generating function $G_S(x, y, t) = \sum_{kl} P_{Skl}(t) x^k y^l$ of the joint probability distribution to find k contacts and l interaction events per time among susceptible individuals P_{Skl}: $\langle l \rangle_S = \sum_{k,l} l \frac{S_{kl}}{S} = G_S^{(0,1)}(1, 1, t)$. The $(0, 1)$ exponent of G_S indicates the orders of the partial derivatives with respect to the first and second argument of G_S (see Table S1).

Summation of S_{kl} and I_{kl} over k and l results in equations for the total number of

susceptible and infected hosts:

$$\dot{S} = -\beta p_{SI} S G_S^{(0,1)}(1,1,t) \tag{2a}$$

$$\dot{I} = \beta p_{SI} S G_S^{(0,1)}(1,1,t) - \gamma I \tag{2b}$$

$$\dot{R} = \gamma I \tag{2c}$$

To close this set of equations we also need to derive equations for p_{SI}, as well as for the probability generating function (PGF) $G_S(x,y,t)$.

We begin by deriving the temporal dynamics of the probabilities for a link starting from a randomly selected susceptible individual to point to a susceptible or infected individual, p_{SS} and p_{SI}, respectively. Following the argument in [1] we write $p_{SS} = M_{SS}/M_S$ and $p_{SI} = M_{SI}/M_S$ to express these probabilities in terms of the total number of links/contacts that connect susceptible and infected hosts (M_{SS}, M_{SI}) and total number of links/contacts of susceptible hosts (M_S) in the network. From this, we get:

$$\dot{p}_{SS} = \frac{\dot{M}_{SS}}{M_S} - \frac{\dot{M}_S}{M_S} p_{SS} \tag{3a}$$

$$\dot{p}_{SI} = \frac{\dot{M}_{SI}}{M_S} - \frac{\dot{M}_S}{M_S} p_{SI} \tag{3b}$$

for which expressions are derived in the following paragraph. From the definition of M_S, we can write the following equation:

$$\dot{M}_S = \sum_{k,l} k \dot{S}_{kl} \tag{4}$$

Substitution of $\dot{S}_{kl}$ from equation (1a) results in

$$\dot{M}_S = -\beta p_{SI} S G_S^{(1,1)}(1,1,t) \tag{5}$$

We then follow the arguments made in an earlier study [1], which rely on the assumption that the number of contacts from susceptible hosts to susceptible, infected and recovered hosts is multinomially distributed with probabilities p_{SI}, p_{SS} and $p_{SR} = 1 - p_{SS} - p_{SI}$. We also assume that the same applies to the number of interaction events/sex acts per time interval. If a node with k contacts has j contacts with susceptible individuals and i contacts with infected individuals its interaction events with susceptible, infected and recovered individuals n_{SS}, n_{SI} and n_{SR}, respectively, are distributed according to

$$\frac{l!}{n_{SS}!n_{SI}!n_{SR}!}\left(\frac{j}{k}\right)^{n_{SS}}\left(\frac{i}{k}\right)^{n_{SI}}\left(\frac{k-j-i}{k}\right)^{n_{SR}} \tag{6}$$

with averages $\langle n_{SS}\rangle = \frac{jl}{k}$,$\langle n_{SI}\rangle = \frac{il}{k}$ and $\langle n_{SR}\rangle = \frac{(k-j-i)l}{k}$. Note that $l = n_{SS} + n_{SI} + n_{SR}$.

The probability that a susceptible node with l interaction events and j, i and $k-i-j$ contacts to susceptible, infected and recovered individuals, respectively, is reached from an infected node, i.e. chosen with a probability proportional to the average number of interaction events with infected nodes ($\langle n_{SI}\rangle = \frac{li}{k}$) is then

$$\frac{P_{kl}\frac{k!}{i!j!k-i-j!}p_{SS}^{j}p_{SI}^{i}p_{SR}^{k-j-i}\langle n_{SI}\rangle}{p_{SI}G^{(0,1)}(1,1)} \overset{\langle n\rangle_{SI}=\frac{li}{k}}{=} \frac{lP_{kl}\frac{(k-1)!}{(i-1)!j!(k-1-(i-1)-j)!}p_{SS}^{j}p_{SI}^{i-1}p_{SR}^{k-j-i}}{G^{(0,1)}(1,1)} \tag{7}$$

Note that the denominator can be derived using the observation that

$$\begin{aligned}
&\sum_{k,l} P_{kl} l p_{SI} \sum_{i,j\leq k} \frac{(k-1)!}{(i-1)!j!(k-1-(i-1)-j)!} p_{SS}^{j} p_{SI}^{i-1} p_{SR}^{k-j-i} \\
&= \sum_{k,l} P_{kl} l p_{SI} (p_{SS} + p_{SI} + p_{SR})^{k-1} && \text{(8a)} \\
&= p_{SI} \sum_{k,l} l P_{kl} = p_{SI} G^{(0,1)}(1,1) && \text{(8b)}
\end{aligned}$$

Therefore, the probability distribution for the contacts and potential interaction events of a node which was chosen proportional to $\langle n_{SI} \rangle = \frac{li}{k}$ is generated by

$$\begin{aligned}
& \frac{\sum_{kl} l P_{kl} y^l \sum_{i,j\leq k} \frac{(k-1)!}{(i-1)!j!(k-1-(i-1)-j)!} (x_S p_{SS})^j (x_I p_{SI})^i (x_R p_{SR})^{k-j-i}}{p_{SI} G^{(0,1)}(1,1)} \\
= & \frac{\sum_{kl} l P_{kl} x_I y^l (x_S p_{SS} + x_I p_{SI} + x_R p_{SR})^{k-1}}{G^{(0,1)}(1,1)} \qquad (9a) \\
= & \frac{x_I y}{x_S p_{SS} + x_I p_{SI} + x_R p_{SR}} \frac{G^{(0,1)}(x_S p_{SS} + x_I p_{SI} + x_R p_{SR}, y)}{G^{(0,1)}(1,1)} \qquad (9b)
\end{aligned}$$

Choosing a node proportional to its average number of interaction events per time ($\langle n_{SI} \rangle = il/k$) instead of the actual number of interaction events (n_{SI}) implies the assumption of a time scale at which $l \gg k$, i.e. a case in which fluctuations around $\langle n_{SI} \rangle$ can be expected to be small. Taking all this together, the average degrees of a susceptible node that was reached from an infected node to susceptible or infected nodes are

$$\begin{aligned}
\delta_{SI}(S) &= \frac{\partial}{\partial x_S} \frac{x_I y}{x_S p_{SS} + x_I p_{SI} + x_R p_{SR}} \frac{G_S^{(0,1)}(x_S p_{SS} + x_I p_{SI} + x_R p_{SR}, y, t)}{G_S^{(0,1)}(1,1)} \Bigg|_{x_S = x_I = x_R = y = 1} \\
&= p_{SS} \left(\frac{G_S^{(1,1)}(1,1,t)}{G_S^{(0,1)}(1,1,t)} - 1 \right) \qquad (10a) \\
\delta_{SI}(I) &= \frac{\partial}{\partial x_I} \frac{x_I y}{x_S p_{SS} + x_I p_{SI} + x_R p_{SR}} \frac{G_S^{(0,1)}(x_S p_{SS} + x_I p_{SI} + x_R p_{SR}, y, t)}{G_S^{(0,1)}(1,1)} \Bigg|_{x_S = x_I = x_R = y = 1} \\
&= p_{SI} \left(\frac{G_S^{(1,1)}(1,1,t)}{G_S^{(0,1)}(1,1,t)} - 1 \right) + 1 \qquad (10b)
\end{aligned}$$

The average number of contacts to susceptible and infected nodes needs to be discounted by one for the number of contacts with infected nodes to take the contact to the source of infection into account (directly considering this in the PGF gives the same result), i.e. the total excess degree of a node that was chosen proportional to its average number of

interaction events with infected individuals $\langle n_{SI} \rangle = il/k$ is $\frac{G_S^{(1,1)}(1,1,t)}{G_S^{(0,1)}(1,1,t)} - 1$.

Bookkeeping of the changes in the numbers of links among susceptible and infected hosts due to the epidemic process results in:

		**Changes due to epidemic spread**
$\dot{M}_{SI} =$	$-\dot{S}(p_{SS} - p_{SI})\left(\frac{G_S^{(1,1)}(1,1,t)}{G_S^{(0,1)}(1,1,t)} - 1\right)$	change in the number of susceptible nodes $\dot{S} = -\beta p_{SI} S G_S^{(0,1)}(1,1,t)$ (cf. equation 2a) due to the epidemic multiplied by their average excess contacts to susceptible and infected nodes (cf. equations 10a and 10b)
	$-\beta \frac{G_S^{(0,1)}(1,1,t)}{G_S^{(1,0)}(1,1,t)} M_{SI}$	discount for link along which the infection spread
	$-\gamma M_{SI}$	link loss due to recovery of infected
$\dot{M}_{SS} =$	$\dot{S} 2 p_{SS}\left(\frac{G_S^{(1,1)}(1,1,t)}{G_S^{(0,1)}(1,1,t)} - 1\right)$	change in the number of susceptible nodes $\dot{S} = -\beta p_{SI} S G_S^{(0,1)}(1,1,t)$ due to the epidemic multiplied by their average excess contacts to infected nodes (bi-directional)

In summary this results in

$$\dot{M}_{SI} = \beta p_{SI} S \left(G_S^{(0,1)}(1,1,t) - G_S^{(1,1)}(1,1,t)\right)(p_{SI} - p_{SS}) - \beta \frac{G_S^{(0,1)}(1,1,t)}{G_S^{(1,0)}(1,1,t)} M_{SI} - \gamma M_{SI} \tag{11a}$$

$$\dot{M}_{SS} = -2\beta p_{SS} p_{SI} S \left(G_S^{(1,1)}(1,1,t) - G_S^{(0,1)}(1,1,t)\right) \tag{11b}$$

which finally leads to

$$\dot{p}_{SI} = \beta p_{SI} p_{SS} \frac{G_S^{(1,1)}(1,1,t) - G_S^{(0,1)}(1,1,t)}{G_S^{(1,0)}(1,1,t)} - \beta p_{SI}(1 - p_{SI}) \frac{G_S^{(0,1)}(1,1,t)}{G_S^{(1,0)}(1,1,t)} - \gamma p_{SI} \tag{12a}$$

$$\dot{p}_{SS} = -\beta p_{SI} p_{SS} \frac{G_S^{(1,1)}(1,1,t) - 2G_S^{(0,1)}(1,1,t)}{G_S^{(1,0)}(1,1,t)}. \tag{12b}$$

To close the set of equations we also need to derive an equation for the probability generating function (PGF) $G_S(x, y, t)$, which corresponds to the probability to find individuals with k contacts and l interaction events (e.g. sex acts) per time interval among susceptible hosts, i.e. P_{Skl}. From the definitions of the PGF and $P_{Skl} = \frac{S_{kl}}{S}$, we obtain

$$\dot{G}_S(x, y, t) = \sum_{k,l} \left(\frac{\dot{S}_{k,l}}{S} - \frac{\dot{S}}{S} P_{Skl} \right) x^k y^l, \tag{13}$$

which results with equations 1a and 2a in

$$\dot{G}_S(x, y, t) = \beta p_{SI} \left(G_S^{(0,1)}(1, 1, t) G_S(x, y, t) - y G_S^{(0,1)}(x, y, t) \right) \tag{14}$$

The probability generating functions $G_I(x, y, t)$ and $G_R(x, y, t)$ can be derived analogously, though they are not needed to close the system of equations:

$$\dot{G}_I(x, y, t) = -\beta p_{SI} \frac{S}{I} \left(G_S^{(0,1)}(1, 1, t) G_I(x, y, t) - y G_S^{(0,1)}(x, y, t) \right) \tag{15a}$$

$$\dot{G}_R(x, y, t) = \beta \frac{I}{R} \left(G_I(x, y, t) - G_R(x, y, t) \right). \tag{15b}$$

B Conditional probabilities and risk groups

Note that $P_{kl} = P(k, l)$ and that for $A \in \{S, I, R\}$ $P_{Akl} = P(k, l|A)$ are the conditional probabilities to have k contacts and l transmission events given that you have status A. This allows for a direct derivation of the average number of contacts $\langle k \rangle_A$ and transmission events $\langle l \rangle_A$ given that you have status A.

$$\langle k \rangle_A = G_A^{(1,0)}(1, 1, t) \tag{16a}$$

$$\langle l \rangle_A = G_A^{(0,1)}(1, 1, t). \tag{16b}$$

Bayes' theorem together with equations 14-15b allows to derive the conditional probabilites for individuals with a certain number of contacts k and interaction events l to be susceptible, infected recovered, i.e. to identify risk groups:

$$P(A|k,l) = \frac{P(A)}{P(k,l)} P(k,l|A) = \frac{P(A)}{P(k,l)} \frac{1}{k!l!} \frac{\partial^{k+l}}{\partial x^k \partial y^l} G_A(x,y,t)_{|x=y=0} \tag{17a}$$

$$P(A|k) = \frac{P(A)}{P(k)} P(k|A) = \frac{P(A)}{P(k)} \frac{1}{k!} \frac{\partial^{k}}{\partial x^k} G_A(x,y,t)_{|x=0,y=1} \tag{17b}$$

$$P(A|l) = \frac{P(A)}{P(l)} P(l|A) = \frac{P(A)}{P(l)} \frac{1}{l!} \frac{\partial^{l}}{\partial y^l} G_A(x,y,t)_{|x=1,y=0}. \tag{17c}$$

C The basic reproductive ratio R_0

The basic reproductive ratio R_0 of a SIR epidemic with transmission rate β and recovery rate γ on a classical (unweighted) network can be derived as [2]

$$R_0 = \frac{g^{(2)}(1)}{g^{(1)}(1)} \int_0^\infty \left(1 - e^{-\beta t}\right) \gamma e^{-\gamma t} dt \tag{18a}$$

$$= \frac{\beta}{\beta + \gamma} \left(\frac{\langle k^2 \rangle}{\langle k \rangle} - 1 \right) \tag{18b}$$

where g is the probability generating function of the network's degree distribution. It is the product of the average excess degree of a node which was reached according to its degree and the transmissibility, i.e. the probability that an infection is spread along a link before recovery (here $\beta/(\beta + \gamma)$). These terms do not factorize in the case where there are l interaction events per node defined through the joint probability distribution P_{kl}. To derive R_0 for this case we first derive the excess degree distribution Q_{kl} of a node that was reached with probability proportional to its activity l and that has k excess contacts and

l interaction events per time interval

$$Q_{kl} = \frac{lP_{(k+1)l}}{G^{(0,1)}(1,1)}, \tag{19}$$

and get, for l interaction events multinomially distributed among the $k+1$ contacts (with probabilities $p_1 = ... = p_{k+1} = \frac{1}{k+1}$, $m_1 + ... + m_{k+1} = l$),

$$\begin{aligned} R_0 &= \sum_{k,l} \int_0^\infty \gamma e^{-\gamma t} \sum_{m_1,...,m_{k+1}} \frac{l!}{m_1!...m_{k+1}!} p_1^{m_1} ... p_{k+1}^{m_{k+1}} \sum_{j=1}^{k} (1 - e^{-m_j \beta t}) Q_{kl} dt \quad (20a) \\ &= \sum_{k,l} \sum_{m_1,...,m_{k+1}} \frac{l!}{m_1!...m_{k+1}!} p_1^{m_1} ... p_{k+1}^{m_{k+1}} \sum_{j=1}^{k} \frac{m_j \beta}{m_j \beta + \gamma} Q_{kl} \quad (20b) \end{aligned}$$

The SI model is trivially included in the SIR network models in the limit $\gamma \to 0$ for which R_0 can be derived from equation (20b):

$$\begin{aligned} R_0 &= \sum_{k,l} \sum_{m_1,...,m_{k+1}} \frac{l!}{m_1!...m_{k+1}!} p_1^{m_1} ... p_{k+1}^{m_{k+1}} k Q_{kl} \quad (21a) \\ &= \sum_{k,l} kl \frac{P_{k+1,l}}{G^{(0,1)}(1,1)} \quad (21b) \\ &= \frac{G^{(1,1)}(1,1) - G^{(0,1)}(1,1)}{G^{(0,1)}(1,1)} \quad (21c) \\ &= \frac{\langle kl \rangle - \langle l \rangle}{\langle l \rangle} = \frac{\langle kl \rangle}{\langle l \rangle} - 1 \quad (21d) \end{aligned}$$

As long as infected individuals stay indefinitely infected, R_0 is not affected by the transmission rate and it measures whether there is a giant connected component in classical random networks. The effect of weighting the contact network using the number of interaction events l is here also noticeable: there are not only contacts required for an infection to spread beyond an individual but also sufficient interaction events.

The basic reproductive ration R_0 for a SIR model on a weighted network can be approximated by

$$\begin{aligned} R_0 &= \sum_{k,l} \sum_{m_1,\dots,m_{k+1}} \frac{l!}{m_1!\dots m_{k+1}!} p_1^{m_1} \dots p_{k+1}^{m_{k+1}} \int_0^\infty \gamma e^{-\gamma t} \sum_{j=1}^{k} (1-e^{-m_j \beta t}) Q_{kl} dt && \text{(22a)} \\ &\approx \sum_{k,l} \sum_{m_1,\dots,m_{k+1}} \frac{l!}{m_1!\dots m_{k+1}!} p_1^{m_1} \dots p_{k+1}^{m_{k+1}} \int_0^\infty \gamma e^{-\gamma t} k (1-e^{-\langle \frac{l}{k} \rangle \beta t}) \frac{l P_{k+1l}}{G^{(0,1)}(1,1)} dt \\ &= \sum_{k,l} kl \frac{P_{k+1l}}{G^{(0,1)}(1,1)} \int_0^\infty \gamma e^{-\gamma t} (1-e^{-\langle \frac{l}{k} \rangle \beta t}) dt \\ &= \frac{\langle \frac{l}{k} \rangle \beta}{\langle \frac{l}{k} \rangle \beta + \gamma} \frac{G^{(1,1)}(1,1) - G^{(0,1)}(1,1)}{G^{(0,1)}(1,1)}. && \text{(22b)} \end{aligned}$$

Note that for the linear case with $P_{l|k} = \delta_{lk}$, we obain $G(x,y) = \sum_{k,l} x^k y^l \tilde{P}_k \delta_{kl} = \sum_k (xy)^k \tilde{P}_k = \bar{G}(xy)$ and $R_0 = \frac{\beta}{\beta+\gamma} \frac{\tilde{G}^{(2)}(1)}{\tilde{G}^{(1)}(1)}$, which is consistent with earlier findings.

D The recovery of the classical equations in the linear case $P_{kl} = P_k \delta_{kl}$

The set of equations for the weighted networks (equations 2a-2c,12a-12b,14) includes the case of classical network epidemic models, i.e. the linear case where $k = l$ or $P_{kl} = P_k \delta_{kl}$. Focusing on the degree distribution among susceptible hosts P_{Sk} with probability generating function $g_S(x)$, the PGF of P_{Skl} is given by $G_S(x,y) = g_S(xy)$. Substitution of

$G_S(x, y)$ by $g_S(xy)$ results in

$$G_S^{(1,0)}(1,1,t) \quad = \quad g'_S(1,t) \tag{23a}$$

$$G_S^{(0,1)}(1,1,t) \quad = \quad g'_S(1,t) \tag{23b}$$

$$G_S^{(1,1)}(1,1,t) \quad = \quad g''_S(1,t) + g'_S(1,t) \tag{23c}$$

and for the time evolution of $G_S(x, y)$:

$$\dot{g}_S(xy,t) = \beta p_{SI} \left(g'_S(1) g_S(xy,t) - xy g'_S(xy,t) \right). \tag{24}$$

Together, this leads to the set of equations for SIR dynamics on a classical configuration type network defined by the degree distribution P_k [1, 3]

$$\dot{S} \quad = \quad -\beta\, p_{SI}\, S\, g'_S(1,t) \tag{25a}$$

$$\dot{I} \quad = \quad \beta\, p_{SI}\, S\, g'_S(1,t) - \gamma\, I \tag{25b}$$

$$\dot{R} \quad = \quad \gamma\, I \tag{25c}$$

$$\dot{p}_{SI} = \beta\, p_{SI}\, p_{SS}\, \frac{g''_S(1,t)}{g'_S(1,t)} - \beta\, p_{SI}\, (1 - p_{SI}) - \gamma\, p_{SI} \tag{25d}$$

$$\dot{p}_{SS} = -\beta\, p_{SI}\, p_{SS} \left(\frac{g''_S(1,t)}{g'_S(1,t)} - 1 \right) \tag{25e}$$

$$\dot{g}_S(x,t) \quad = \quad \beta p_{SI} \left(g'_S(1,t) g_S(x,t) - x g'_S(x,t) \right). \tag{25f}$$

E Network segregation and the limiting case $P_{kl} = P_k\delta_{\langle l\rangle l}$ (constant case)

The analytical approximation assumes that an individual distributes his/her interaction events l multinomially among his/her k contacts and is infected at a rate proportional to his/her average number of potential transmission events with i infected contacts. This averaging implies the choice of a time scale such that $\langle l\rangle > \langle k\rangle$. This leads to an unrealistic network segregation in some artificial networks, specifically for $\langle l\rangle \gg \langle k\rangle$, as the weights of an individual's contacts level at about l/k which enforces contacts only between individuals with (almost) identical l/k. This network segregation affects epidemiological dynamics. As the analytical approach is node-centric it does not consider the constraints on half-contacts to match half-contacts of similar weight. In consequence, the change in epidemic dynamics due to networks segregation cannot be seen in the analytical approach.

The effect is particularly pronounced if we have a network with a heterogeneous degree distribution (which corresponds to a case where many individuals have only one contact) combined with a constant number of interaction events per individual. Degree one nodes have only one contact to assign their interaction events to, which leaves their contacts on average already with twice the weight seen in individuals with two contacts (i.e. $\langle l\rangle$ for $k = 1$ vs. $\frac{\langle l\rangle}{2}$ for $k = 2$). This weight separation leads to a situation that almost only allows contacts among individuals with a single contact, i.e. monogamous couples (contact or degree assortativity). Therefore, individuals with one contact can only be infected if

their partner is initially infected but not later on through the epidemic process because they are not connected to the giant component of the network. In the case of a constant number of interaction events per individual $\langle l\rangle$, the analytical approach breaks into independent equations for all k classes with $\langle l\rangle_S = \langle l\rangle$ in which epidemic prevalence grows at the same rate:

$$\dot{S}_{k\langle l\rangle} = -\beta p_{SI}\langle l\rangle S_{k\langle l\rangle} \tag{26a}$$

$$\dot{I}_{k\langle l\rangle} = +\beta p_{SI}\langle l\rangle S_{k\langle l\rangle} - \gamma I_{k\langle l\rangle} \tag{26b}$$

$$\dot{R}_{k\langle l\rangle} = \gamma I_{k\langle l\rangle}. \tag{26c}$$

Due to the network segregation, epidemic prevalence is reduced in these networks at least by a factor proportional to the fraction of nodes with a single contact as compared to the standard result of the analytical approach. Again, this is because in these type of networks the single-contact nodes do not participate in the epidemic process.

F Agreement between approximations and simulations

We evaluate the agreement between simulations and approximations based on the evolution of infected from time $t = 0$ to time $t = 1500$ expressed in arbitrary units (see main text for parameter values and details). For each network type, we compare analytical approximations (denoted $y_{raw}(t)$, $y_{rem}(t)$ and $y_{emp}(t)$) to the mean of 2000 simulation replicates at each point of time t (denoted $x(t)$). $y_{raw}(t)$, $y_{rem}(t)$ and $y_{emp}(t)$ correspond respectively to raw approximations, approximations when nodes with one partner are removed and

approximations based on the empirical distributions.

The level of agreement between simulations and approximations is assessed with cross-correlation analysis and linear regression:

- *Cross correlation analysis.* We calculate $\tau^* = \max_\tau \mathcal{C}(x, y, \tau)$ and $\mathcal{C}^* = \mathcal{C}(x, y, \tau^*)$, where $\mathcal{C}(x, y, \tau)$ is the sample cross correlation coefficient between time series x (set as the reference) and y for a time lag set to τ (function *ccf* in R). τ takes values in $[-1500, 1500]$ since time has set in $[0, 1500]$. τ^* is the value of τ that maximizes the value of $\mathcal{C}(x, y, \tau)$, denoted $\mathcal{C}^*$. In other words, τ^* quantifies the point in time where times series x and y are optimally correlated. A perfect agreement between simulations and data implies $\tau^* = 0$ and $\mathcal{C}^* = 1$.

- *Linear regression.* We fit of the model $y(t) = ax(t)$ (function *lm* in R). The match between simulations and approximations is optimal when $a = 1$, p-value< 0.001 and $R^2 = 1$.

G Captions of the Supplementary Figures

Figure S1: Epidemic SIR dynamics on the network as presented in Fig. 3 of the main manuscript. Transmission probability per sex act is also $\beta = 0.01$ but recovery can occur at a rate $\gamma = 0.004$ per 4 weeks, i.e. parameters corresponding to Fig. 2 of the main manuscript. Different from the SI dynamics shown in Fig.3 of the main manuscript hosts may recover and do not spread infection indefinitely.

Figure S2: Epidemic incidence or rate of infection $\beta p_{SI} S G_S^{(0,1)}(1, 1, t) = \beta p_{SI} S \langle l \rangle_S$ (cf. equation 2b) for SI dynamics (grey line) and SIR dynamics (dark grey line) on the network as presented in Fig. 3 of the main manuscript.

Figure S3: Relationship between a person's total number of sex acts and number of partners derived from the NATSAL data. In Panel A, we plot the self-reported number of sex acts over the last 4 weeks *vs.* the self-reported number of sexual partners over the last 4 years. In Panel B, we plot the self-reported number of sex acts over the last 4 weeks *vs.* the self-reported number of sexual partners over the last 3 months. In Panel C, we plot the self-reported number of sex acts over the last 7 days *vs.* the self-reported number of sexual partners over the last 3 months. In all three cases, the data do not support a linear relationship (the number of sex acts per partner decreases with the number of partners/contacts).

H Captions of the Supplementary Tables

Table S1: Model notations

Table S2: Agreement between approximations and simulations

References

[1] Volz E (2008) SIR dynamics in random networks with heterogeneous connectivity. J Math Biol 56: 293-310.

[2] Durrett R (2007) Random graph dynamics. Cambridge University Press.

[3] Kamp C (2010) Untangling the interplay between epidemic spread and transmission network dynamics. PLoS Comput Biol 6: e1000984.
